# Irreversibility Interpretation and MHD Mixed Convection of Hybrid Nanofluids in a 3D Heated Lid-Driven Chamber

**DOI:** 10.3390/nano12101747

**Published:** 2022-05-20

**Authors:** Houssem Laidoudi, Aissa Abderrahmane, Abdulkafi Mohammed Saeed, Kamel Guedri, Wajaree Weera, Obai Younis, Abed Mourad, Riadh Marzouki

**Affiliations:** 1 Laboratory of Sciences and Marine Engineering, Faculty of Mechanical Engineering, University of Science and Technology of Oran—Mohamed Boudiaf, Oran 31000, Algeria; houssem.laidoudi@univ-usto.dz; 2 Laboratoire de Physique Quantique de la Matière et Modélisation Mathématique (LPQ3M), University of Mascara, Mascara 29000, Algeria; a.aissa@univ-mascara.dz (A.A.); mourad.abed@univ-mascara.dz (A.M.); 3 Department of Mathematics, College of Science, Qassim University, P.O. Box 6644, Buraydah 51452, Saudi Arabia; abdulkafi.ahmed@qu.edu.sa; 4 Department of Mathematics, Hodeidah University, P.O. Box 3114, Al-Hudaydah 207416, Yemen; 5 Mechanical Engineering Department, College of Engineering and Islamic Architecture, Umm Al-Qura University, P.O. Box 5555, Makkah 21955, Saudi Arabia; kmguedri@uqu.edu.sa; 6 Department of Mathematics, Faculty of Science, Khon Kaen University, Khon Kaen 40002, Thailand; 7 Department of Mechanical Engineering, College of Engineeing at Wadi Addwaser, Prince Sattam Bin Abdulaziz University, P.O. Box 173, Al-Kharj 11942, Saudi Arabia; oubeytaha@hotmail.com; 8 Chemistry Department, College of Science, King Khalid University, P.O. Box 394, Abha 61413, Saudi Arabia; rmarzouki@kku.edu.sa

**Keywords:** convection, irreversibility analysis, MHD, hybrid nanofluid, GFEM

## Abstract

This paper presents a numerical simulation of a magneto-convection flow in a 3D chamber. The room has a very specific permeability and a zigzag bottom wall. The fluid used in this study is Al_2_O_3_-Cu/water with 4% nanoparticles. The Galerkin finite element technique (GFEM) was developed to solve the main partial equations. The hybrid nanofluid inside the container is subjected to the horizontal motion of the upper wall, an external magnetic field, and a thermal buoyancy force. The present numerical methodology is validated by previous data. The goal of this investigation was to understand and determine the percentage of heat energy transferred between the nanofluid and the bottom wall of the container under the influence of a set of criteria, namely: the movement speed of the upper wall of the cavity (Re = 1 to 500), the amount of permeability (Da = 10^−5^ to 10^−2^), the intensity of the external magnetic field (Ha = 0 to 100), the number of zigzags of the lower wall (N = 1 to 4), and the value of thermal buoyancy when the force is constant (Gr = 1000). The contours of the total entropy generation, isotherm, and streamline are represented in order to explain the fluid motion and thermal pattern. It was found that the heat transfer is significant when (N = 4), where the natural convection is dominant and (N = 2), and the forced convection is predominant.

## 1. Introduction

Mixed convection is a complicated phenomenon in thermal transfer processes, and it is generated owing to the interplay of shear flow induced by a moving interface and buoyancy-driven flow. It helps in boosting heat transfer and flow mixing characteristics. Apart from this, an increase in the ratio of heat transfer is a vital concern owing to the sparkling improvements in current technologies. Among the most important thermal transfer processes, mixed convection heat transfer is massively confronted in many engineering and industrial investigations such as heat exchangers, the cooling of microelectronic and electronic devices, solar collectors, pharmaceutical processes, etc.

Engineers and researchers have assembled to build revolutionary thermal transport fluids by incorporating very fine particles with regular cooling liquids called nanofluids. Nanofluids feature specific qualities, including greater heat conductivity, stability, the lowest dragging force, etc. Therefore, various studies have been undertaken on double-diffusion convection inside a lid-driven container loaded with normal fluids or nanofluids with varied temperature settings. A few relevant past studies have been provided here [1,2,3,4]. Zakaria et al. [5] explored the magneto-convection flow generated by the lid in a chamber with two curved edges; here, the bottom side was partly heated and loaded with a hybrid nanofluid composed of Al2O3-Cu/water. The authors observed that the composition ratio (Al_2_O_3_ 75%, Cu 25%) offered the greatest values of both the total entropy production and the mean Nusselt number. Furthermore, the overall irreversibility and heat transfer diminish with rising Ha and declining Ri. Haiying et al. [6] modeled the laminar regime’s mixed flow and nanofluid’s heat transfer within an enclosed semi-elliptic lid-driven chamber. The collected findings suggest that introducing a greater volume percentage of nanoparticles at small values of Richardson numbers contributes to the augmentation of heat transfer and the mean Nusselt number. An-Yang et al. [7] provided a very accurate wavelet-homotopy resolution for a double-diffusion convection hybrid nanofluid flow inside a tilted squared lid-driven enclosure. They determined that the hybrid nanofluid is better than regular heat transfer fluids for heat transfer improvement and is comparable to nanofluids, but it might be simpler and cheaper. Jakeer et al. [8] evaluated the influence of a hot barrier location on a magneto-hybrid nanoliquid flow in a porous chamber under the effect of a lid-driven flow with a Cattaneo–Christov heat flux. Based on their conclusions, the thermo-fluidic coefficients in the direction of the displacement of the heated obstruction are discovered to have an essential effect. Manchanda et al. [9] assessed the double-diffusion convection flow computationally inside a double lid-driven rectangular chamber with a heated triangular obstruction.

The Lorentz force connected with fluids that carry electric charges has garnered substantial study owing to its relevance in engineering applications. In the event of double diffusion, which refers to flow owing to moving walls, the involvement of a magnetic field might impact the fluid flow and heat transfer processes. It is of primary interest to investigate the features of the energy transport of the double diffusion of nanofluids inside lid-driven enclosures with the impact of magnetic forces for the optimal development of engineering devices [10,11,12]. Bakar et al. [13] employed a finite volume approach to uncover the considerable influence of the Lorentz force on the flow and thermal field inside a lid-driven rectangular enclosure. They finally demonstrated that both the heat transfer and flow convection rate diminished with higher Ha. Khanafer et al. [14] utilized the commercial program ADINA to explore mixed convective heat transfer inside a lid-driven enclosure with a spinning obstacle. It was noted that the mean Nusselt number is related to the direction of the rotational velocity and increases with a rise in the angular velocity. Ali et al. [15] explored the double-diffusion convection in a nanoliquid loaded-cavity generated by thermal buoyancy force. They also included impacts from an external magnetic field, a sliding wall and a rotating flat plate. According to their data, the optimum heat transfer is guaranteed when the movement of the spinning plate is the same as the movement of the lid wall. Furthermore, excellent heat transfer performance is attained when using nanoparticles with a 5 percent concentration. The heat transfer rate was found to be 123.02 percent greater than that for simple fluid. Ghasemi et al. [16] computationally explored the MHD convective flow of nanoliquid in a cubical chamber containing lid-driven walls. The acquired findings indicated that nano additives improve heat transfer, whereas a magnetic field decreases the proposed convective process. Geridonmez et al. [17] explored mixed convective flow in a lid-driven chamber. The influence of a magnetic field was partially applied. They observed that the fluid movement and heat transfer are delayed when the Lorentz force grows. Hussain et al. [18] examined the influence of fins and Lorentz force with nanoliquid in both double-lid-driven and single-lid-driven chambers. The collected data determined that the velocity of flow and convective heat transfer decline with the rise in Ha and Ri numbers in all conditions.

Many scholars have investigated double-diffusion convection in a porous space under a variety of settings, such as lid-driven cavities filled with nanofluid. Gutt et al. [19] explored lid-driven chamber issues using the Darcy –Brinkman model numerically and analytically. It was noticed that an increment in space permeability resulted in moving the vorticity center and reducing the value of φ max (the stream function). Eren Çolak et al. [20] numerically studied mixed convection inside a lid-driven container with a partly heated porous block. It was noticed that space permeability might be utilized to regulate the counter-rotating zone generation in the space under specified geometrical conditions. Astanina et al. [21] explored double porous sections’ lid-driven enclosure issues, and they employed nanofluid as a thermal transfer medium. They observed that the impact of the layer thickness of porous sections on heat transfer and flow is non-linear, and a Darcy number of the lowest porous layer has a limited effect on the hydrodynamic pattern. Lei et al. [22] studied double diffusion with porous fins issues and numerically explored the lid-driven chamber scenario. It was noticed that a rise in Darcy number improves the heat transfer; however, the amplification is restricted, and beyond a given space permeability, heat transfer diminishes. Dadavi et al. [23] explored a lid-driven container issue under a double-diffusion flow and coarse porous material. They concluded that a porous medium decreases the mean value of Nu under Re = 1000 settings.

Flow and heat transfer from non-linear surfaces are regularly found in various engineering utilizations. One of the potential approaches for heat transfer enhancement in cavities is to employ irregular (zigzag) active walls rather than smooth ones [24,25,26,27]. Masoud Ali et al. [28] examined the microchannel heat sink topologies’ dynamic flow and thermal characteristics using zigzag, rectangular, and twisted fins. They discovered that the zigzag fin and 3% of Al_2_O_3_ nanoparticles give the highest heat efficiency, with a 60% higher value of Nu and 15% greater efficiency of second law than without fins and with ordinary liquid cooling (water). A. Alnaqi et al. [29] examined the heat transfer performance in hybrid nanofluids utilized to cool micro-heat sink with the zigzag surfaces of micro-channels exposed to a continuous heat source. The findings indicated that raising the velocity enhances the heat generation from the MHS, whereas extending the length of the zigzag of the channel enhances the temperature distribution from the MHS’s surface, and therefore improves the evacuation of thermal energy, which is related to a rise in the pressure difference (∆P) of the passing fluid. Oudina et al. [30] assessed the influences of convection and entropy production on a hybrid nanofluid within a trapezoidal chamber with a zigzagged surface and magnetized system. The findings demonstrated that an intervening magnetic field greatly impacts the generated flow of the working fluid, and the heat efficiency of the chamber is enhanced by increasing the Ra and Ha values. Abderrahmane et al. [31] presented a numerical experience of double-diffusion heat transfer in a 3D triangular porous container with a zigzag wall and a revolving obstacle in the center of the studied area. The results demonstrate that for obtaining optimal rates of heat transfer in hybrid nanofluid in a 3-D triangular porous compartment equipped with a rotating obstacle and exposed to an external magnetic field, a Hartmann number of <0, Darcy number of 10^−3^, rotation speed of >500 of the cylinder in the flow direction, and one zigzag on the hot surface are advised. Chabani et al. [32] numerically investigated thermal transmission using a Cu-TiO_2_/EG hybrid nanofluid within a porous annular zone between a zigzagged triangle and various obstacles and in the existence of an inclined magnetic field. From the findings, it was obtained that by excluding the Ha number, which decelerates the flow rate, the Rayleigh number, the nanofluid volumetric fraction, and the rotational speed of the obstacle have a beneficial influence on the thermal transmission rate. 

Since the use of nanofluids with wall zigzags helps to improve heat transfer, we decided through this paper to present new data on this issue. Accordingly, this paper simulates the motion of a hybrid nanofluid inside a three-dimensional chamber immersed in a magnetic field. The interior space of the cavity is permeable to the fluids, while all walls are impermeable. As for the cavity walls, the upper wall moves horizontally at a constant speed, while the lower zigzag wall is stationary.

The results of this work help expand the theoretical facts about nanofluids and provide some values for the coefficient that helps in the achievement and development of heat exchangers and cooling systems.

## 2. Mathematical Model and the Study Configuration

**Figure 1:**
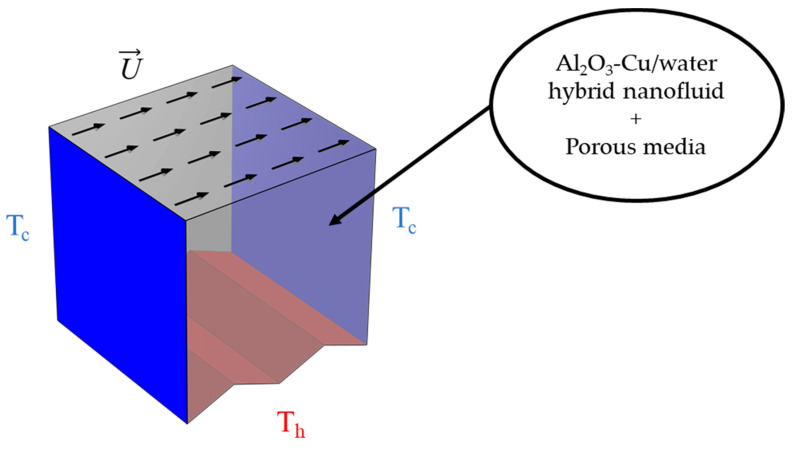
The physical domain and boundary conditions.

The values of the thermophysical properties of the elements of the nanofluid are listed in Table 1. The considered configuration of this work is illustrated in Figure 1 as a 3D-zigzag porous cavity containing a nanofluid with a magnetic force applied along the positive y- and z-directions. All walls are assumed to be adiabatic and no-slip, except the zigzag wall, which is considered at the hot temperature denoted (Th), and the front wall is at the cold temperature denoted (Tc). The zigzag wall is taken to be the main geometry influencer, which will have different undulations (various peak numbers, N = 4, 2, and 1). The top walls are moving in opposite directions with a constant speed of U.

### 2.1. Mathematical Model

By assuming that the study is within a 3D porous cavity and the selected liquid is a Newtonian-incompressible fluid undergoing a laminar regime, the governing equations are as follows [35]:

The conservation equations are:

(1) $$\frac{\partial U}{\partial X}+\frac{\partial V}{\partial Y\;}+\frac{\partial W}{\partial Z}=0$$

The momentum equations, along with the three directions, are:

(2a) $$\begin{array}{l} \frac{\rho_{nf}}{\rho_{f}}\left[\frac{U}{\varepsilon^{2}}\frac{\partial U}{\partial X}+\frac{V}{\varepsilon^{2}}\frac{\partial U}{\partial Y}+\frac{W}{\varepsilon^{2}}\frac{\partial U}{\partial Z}\right]=-\frac{\rho_{nf}}{\rho_{f}}\frac{\partial P}{\partial X}+\frac{1}{Re}\frac{1}{\varepsilon }\frac{\mu_{hnf}}{\mu_{f}}\left(\frac{\partial U}{\partial X}+\frac{\partial U}{\partial Y}+\frac{\partial U}{\partial Z}\right)\\ -\frac{\mu_{hnf}}{\mu_{f}ReDa}U-\frac{\rho_{hnf}}{\rho_{f}}\frac{0.55}{\sqrt{Da}}\sqrt{U^{2}+V^{2}+W^{2}}\;U \end{array}$$

(2b) $$\begin{array}{l} \frac{\rho_{nf}}{\rho_{f}}\left[\frac{U}{\varepsilon^{2}}\frac{\partial V}{\partial X}+\frac{V}{\varepsilon^{2}}\frac{\partial V}{\partial Y}+\frac{W}{\varepsilon^{2}}\frac{\partial V}{\partial Z}\right]=-\frac{\rho_{hnf}}{\rho_{f}}\frac{\partial P}{\partial Y}+\frac{1}{Re}\frac{1}{\varepsilon }\frac{\mu_{hnf}}{\mu_{f}}\left(\frac{\partial V}{\partial X}+\frac{\partial V}{\partial Y}+\frac{\partial V}{\partial Z}\right)\\ -\frac{\mu_{nf}}{\mu_{f}ReDa}V-\frac{\rho_{hnf}}{\rho_{f}}\frac{0.55}{\sqrt{Da}}\sqrt{U^{2}+V^{2}+W^{2}}V-\frac{\sigma_{hnf}}{\sigma_{f}}Ha^{2}\;\frac{V}{\varepsilon } \end{array}$$

(2c) $$\begin{array}{l} \frac{\rho_{hnf}}{\rho_{f}}\left[\frac{U}{\varepsilon^{2}}\frac{\partial W}{\partial X}+\frac{V}{\varepsilon^{2}}\frac{\partial W}{\partial Y}+\frac{W}{\varepsilon^{2}}\frac{\partial W}{\partial Z}\right]=-\frac{\rho_{hnf}}{\rho_{f}}\frac{\partial P}{\partial Z}+\frac{1}{Re}\frac{1}{\varepsilon }\frac{\mu_{hnf}}{\mu_{f}}\left(\frac{\partial W}{\partial X}+\frac{\partial W}{\partial Y}+\frac{\partial W}{\partial Z}\right)\\ \;\;\;-\frac{\mu_{nf}}{\mu_{f}ReDa}W-\frac{\rho_{hnf}}{\rho_{f}}\frac{0.55}{\sqrt{Da}}\sqrt{U^{2}+V^{2}+W^{2}}\;W+\frac{{\left(\rho \beta \right)}_{hnf}}{{\left(\rho \beta \right)}_{f}}Ri\theta -\frac{\sigma_{hnf}}{\sigma_{f}}Ha^{2}\;\frac{W}{\varepsilon } \end{array}$$

where the latest term in Equations (2b) and (2c) are Lorentz forces.

The heat equation is:

(3) U∂θ∂X+V∂θ∂X+W∂θ∂Z=(ρcP)f(ρcP)hnfkeffkf1RePr∂2θ∂X2+∂2θ∂Y2+∂2θ∂Z2

where $k_{eff}=(1-\varepsilon )k_{s}+\varepsilon k_{nf}$ (*k_s_* is referred to the solid thermal conductivity for the matrix of the porous layer, *k_s_* = 0.78 W/m.K and ε = 0.37),

$X,Y,Z=\frac{x,y,z}{L}$, $U,V,W=\frac{\left(u,v,w\right)L}{\alpha_{nf}}$, $\theta =\frac{T-T_{c}}{T_{h}-T_{c}}$, $P=\frac{pL^{2}}{\rho_{nf}\alpha_{fl}^{2}}$, $\mathrm{Pr}=\frac{v_{f}}{\alpha_{f}}$, $Da=\frac{K}{L^{2}}$$Ra=\frac{g\beta_{f}(T_{h}-T_{c})L^{3}}{\alpha_{f}v_{f}}$, $Ha=LB\sqrt{\frac{\sigma_{nf}}{\mu_{nf}}}$, ε is the porosity, and $Ri=\frac{Gr}{Re^{2}}$.

In the present study, the thermophysical properties of the hybrid nanofluid [36,37,38,39] are considered as follow:

- Dynamic viscosity: $\mu_{hnf}=\mu_{f}{\left(1-\phi_{Al_{2}O_{2}}-\phi_{Cu}\right)}^{-2.5}$;
- Density: $\rho_{hnf}=\phi_{Al_{2}O_{3}}\rho_{Al_{2}O_{3}}+\phi_{\mathrm{Cu}}\rho_{\mathrm{Cu}}+\left(1-\phi_{hnf}\right)\rho_{f}$;
- Specific heat: ${\left(\rho C_{p}\right)}_{hnf}=\phi_{Al_{2}O_{3}}{\left(\rho C_{p}\right)}_{Al_{2}O_{3}}+\phi_{Cu}{\left(\rho C_{p}\right)}_{Cu}+\left(1-\phi_{hnf}\right){\left(\rho C_{p}\right)}_{f}$;
- Thermal expansion coefficient: $\beta_{hnf}=\varphi_{1}\beta_{s1}+\varphi_{2}\beta_{s2}\left(1-\varphi_{hnf}\right)\beta_{f}$,

where $\varphi_{hnf}=\varphi_{1}+\varphi_{2}$;

- Electrical conductivity:$\frac{\sigma_{hnf}}{\sigma_{f}}=\left\{\frac{\phi_{Al_{2}O_{3}}\sigma_{Al_{2}O_{3}}+\phi_{Cu}\sigma_{Cu}}{\phi_{Al_{2}O_{3}}+\phi_{Cu}}+2\sigma_{f}+2\left(\phi_{Al_{2}O_{3}}\sigma_{Al_{2}O_{3}}+\phi_{Cu}\sigma_{Cu}\right)-2\left(\phi_{Al_{2}O_{3}}+\phi_{Cu}\right)\sigma_{f}\right\}\times {\left\{\frac{\phi_{Al_{2}O_{3}}\sigma_{Al_{2}O_{3}}+\phi_{Cu}\sigma_{Cu}}{\phi_{Al_{2}O_{3}}+\phi_{Cu}}+2\sigma_{f}-\left(\phi_{Al_{2}O_{3}}\sigma_{Al_{2}O_{3}}+\phi_{Cu}\sigma_{Cu}\right)+\left(\phi_{Al_{2}O_{3}}+\phi_{Cu}\right)\sigma_{f}\right\}}^{-1}$; and
- Thermal conductivity: $\frac{k_{hnf}}{k_{f}}=\left\{\frac{\phi_{Al_{2}O_{3}}k_{Al_{2}O_{3}}+\phi_{Cu}k_{Cu}}{\phi_{Al_{2}O_{3}}+\phi_{Cu}}+2k_{f}+2\left(\phi_{Al_{2}O_{3}}k_{Al_{2}O_{3}}+\phi_{Cu}k_{Cu}\right)-2\left(\phi_{Al_{2}O_{3}}+\phi_{Cu}\right)k_{f}\right\}\times$ ${\left\{\frac{\phi_{Al_{2}O_{3}}k_{Al_{2}O_{3}}+\phi_{Cu}k_{Cu}}{\phi_{Al_{2}O_{3}}+\phi_{Cu}}+2k_{f}-\left(\phi_{Al_{2}O_{3}}k_{Al_{2}O_{3}}+\phi_{Cu}k_{Cu}\right)+\left(\phi_{Al_{2}O_{3}}+\phi_{Cu}\right)k_{f}\right\}}^{-1}$.

### 2.2. Boundary Conditions

The following table lists the boundary conditions of the presented study:



**Thermal Condition**

**Velocity Condition**
The left wall

θ=0



U,V,W=0

The right wall

θ=0



U,V,W=0

The top walladiabatic

U=1,V,W=0

The lower wall

θ=1



U,V,W=0



### 2.3. The Total Entropy Generation $S_{tot}$

The dimensionless form of the total entropy generation $S_{TOT}$ is expressed as follows [40]:

(4) $$S_{TOT}=S_{HT}+S_{FF}+S_{MF}$$

where

(5) $$S_{HT}=\frac{k_{hnf}}{k_{fluid}}\left[{\left(\frac{\partial \theta }{\partial X}\right)}^{2}+{\left(\frac{\partial \theta }{\partial Y}\right)}^{2}+{\left(\frac{\partial \theta }{\partial Z}\right)}^{2}\right],$$

(6) $$\begin{array}{l} S_{FF}=\frac{\mu_{hnf}}{\mu_{fluid}}\varphi \left[\begin{array}{l} 2{\left(\frac{\partial U}{\partial X}\right)}^{2}+2{\left(\frac{\partial V}{\partial Y}\right)}^{2}+2{\left(\frac{\partial W}{\partial Z}\right)}^{2}\\ +{\left(\frac{\partial U}{\partial Y}+\frac{\partial V}{\partial X}\right)}^{2}+{\left(\frac{\partial W}{\partial Y}+\frac{\partial V}{\partial Z}\right)}^{2}\\ +{\left(\frac{\partial U}{\partial Z}+\frac{\partial W}{\partial X}\right)}^{2}+\frac{U^{2}+V^{2}+W^{2}}{Da} \end{array}\right] \end{array}$$

and

(7) $$S_{MF}=\varphi \frac{\sigma_{hnf}}{\sigma_{fluid}}\frac{Ha^{2}}{\varepsilon }\left(W^{2}+V^{2}\right),$$

where $\varphi =\frac{\varepsilon \mu_{nf}T_{0}}{k_{eff}}{\left(\frac{\alpha_{nf}}{L\Delta T}\right)}^{2}$, with $T_{0}=\frac{T_{h}+T_{c}}{2}=0.5$ and $\Delta T=T_{h}-T_{c}$.

The dimensionless form of the Bejan number is as follows:

(8) $$Be=\frac{S_{HT}}{S_{TOT}}$$

The local and average Nusslet numbers are valued as follows:

Locale Nusslet,

(9) $$Nu=-\frac{k_{eff}}{k_{fl}}\frac{\partial \theta }{\partial S}\;;\;\mathrm{and}$$

Average Nusslet,

(10) $$\bar{Nu}=\frac{1}{S}\int_{0}^{S}Nu\;dxdz$$

## 3. Numerical Method and Validation

**Figure 2:**
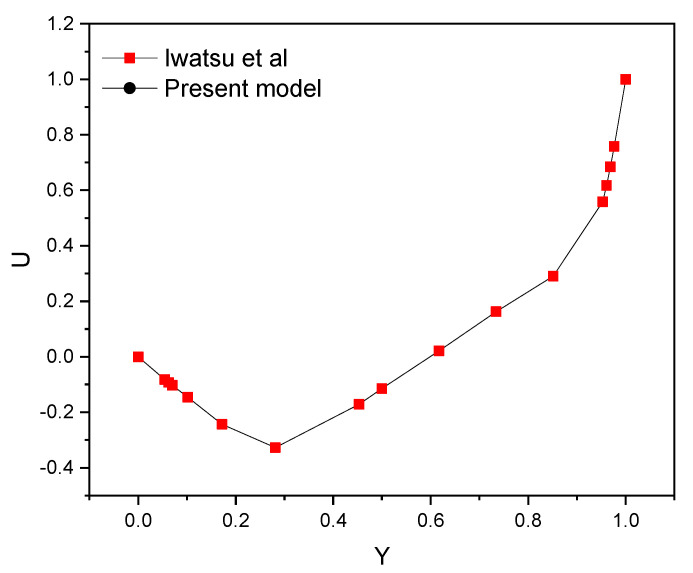
Comparison of velocity profile (current study vs. Iwatsu et al.’s data of [42]).

In order to calculate the Equations (4)–(10), the main partial Equations (1)–(3) were numerically solved using the suitable boundary conditions. Solving the equations was carried out using the Galerkin weighted residual finite element [41]. Non-uniform triangular components were obtained to discretize the issuing domain. The triangular elements with six nodes were adopted to construct the finite element equations. The main partial differential equations were covered in a system of integral equations using he Galerkin weighted residual approach. Gauss’s quadrature method was then used to solve each term. Generally, the purpose is to determine an algebraic system appropriate to the boundary conditions. Different grids were used for the mesh dependence study. For the present simulations, a grid of 511,449 elements was selected (Table 2). In order to test the present numerical approach of the mathematical methodology of the code, the velocity profile inside a room with cylinders was determined and compared (Figure 2) with Iwatsu et al.’s [42] work.

## 4. Results and Discussion

This paper intends to present new results concerning the dynamic behavior of a hybrid nanofluid in a 3D container under the impact of Lorentz and thermal buoyancy forces. An understanding the hybrid nanofluids’ dynamic behavior and its retroactive effect on heat transfer was achieved by analyzing and interpreting the pathlines, distribution of isotherms, and total energy. Further, the interior of the chamber was characterized by a specific permeability to fluids.

The pertinent parameters for the present study are as follows: Reynolds number (Re = 1, 10, 100, and 500), Darcy number (Da = 10^−5^ to 10^−2^), Hartmann number (Ha = 0 to 100), and, finally, the number of zigzags in the bottom wall of the cavity (N = 1, 2, 3, and 4). In addition to this, we mentioned that 4% was the percentage of the nanoparticles in the fluid.

This flow indices a forced-type of convection heat transfer. Before we proceed to presenting and analyzing the results, we would like to highlight the following: the upper wall of the chamber moves horizontally, and this is what moves the adjacent fluid layers with it, and accordingly, a forced flow is created inside the chamber. On the other hand, the fact that the bottom wall is hot and the nanofluid in the room is cold created a difference in density distribution and, thus, the formation of an internal natural flow accompanied by the natural convection heat transfer. Together, the heat transfer is of mixed type. Since the value of the Grashof number in this work is (Gr = 1000), this means that the natural convection is predominant for Re = 1 and 10, whereas, for Re = 100 and 500, the forced convection is predominant.

**Figure 3:**
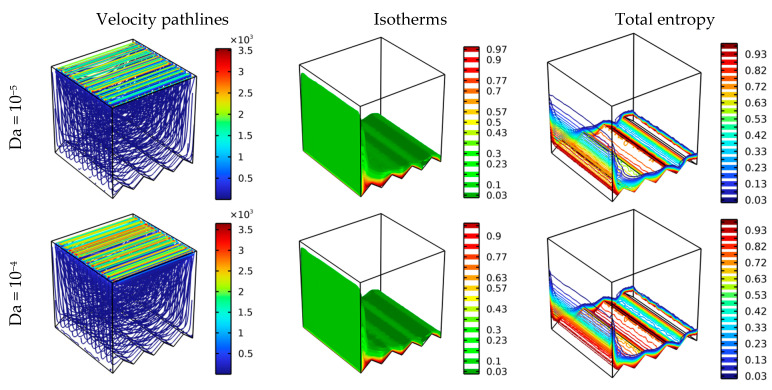

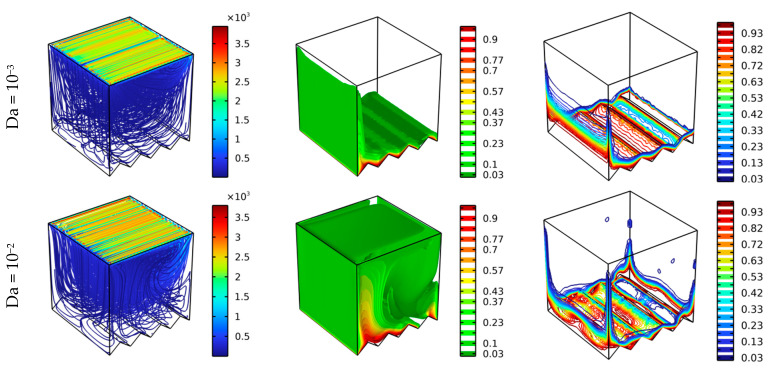
Velocity pathlines, isotherms, and total entropy for different Da at various scenarios for Ha = 0, φ = 0.04, and Re = 100.

Figure 3 represents the effect of the Darcy number value on each of the velocity pathlines and the thermal and total entropy distributions inside the studied container. The value of the Reynolds number is 100, and the number of zigzags is 4 (N = 4). The effect of the Lorenz force is nil in this case as Ha = 0. Through Figure 3, it is clear that raising the value of the Darcy number makes the permeability of the medium greater and moves the fluid particles faster and more easily. The movement of the flow is circular; this is what is indicated in the contours of pathlines. It is also noted that due to the shape of the zigzags on the bottom side, the flow was divided into two parts for Da = 10^−2^. The distribution of isotherms confirms the previous observation. The temperature gradient is higher on the right side of the bottom wall than on the left side, which confirms that the evacuation of heat on the right side is greater. The ease of movement of the fluid particles inside the container due to the improvement of the medium’s permeability augmented the total entropy by augmenting the value of the number of (Da).

**Figure 4:**
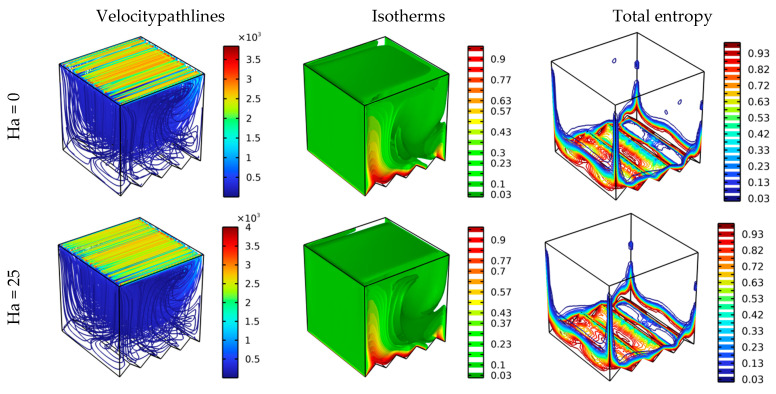

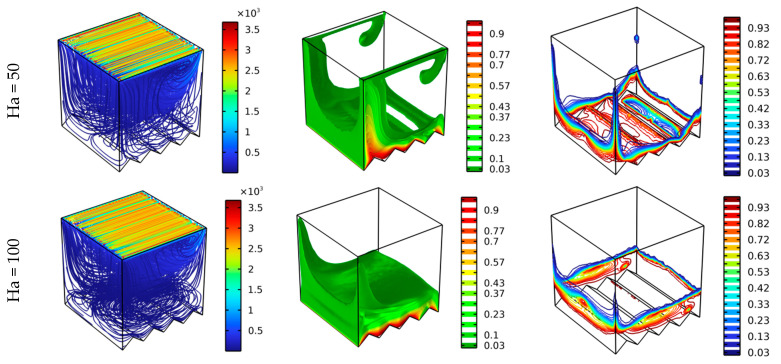
Distribution of the velocity pathlines, isotherm, and total entropy for different Ha values at various scenarios for Da = 10^−2^, φ = 0.04, and Re = 100.

Figure 4 mainly depicts the effects of the Hartmann number value on the contours of the pathline, isotherm, and total entropy for the constant values of the Reynolds number (Re = 100) and zigzag number (N = 4). Through the streamlines of Figure 4, it is clear that raising the value of Ha from 0 to 100 negatively affects the movement of the fluid particles; that is, the movement becomes less uniform, reducing the inertia of the flow. This is, of course, due to the emergence of the Lorentz force, which hinders the direction of the movement of the flow. It is also noted that this movement negatively affected the heat transfer between the bottom side and the nanofluid, and this is what the isotherms in Figure 4 show. That is, there is a decrease in the temperature gradient as the value of *Ha* increases. Further, Figure 4 shows a decrease in total entropy generation due to a decrease in flow velocity due to the negative effect of the Lorentz force.

**Figure 5:**
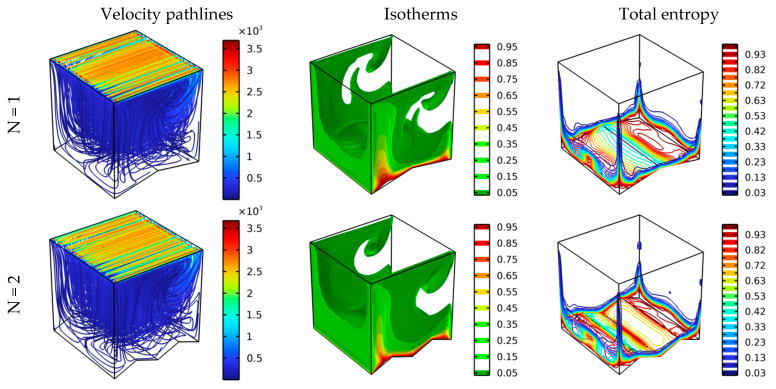

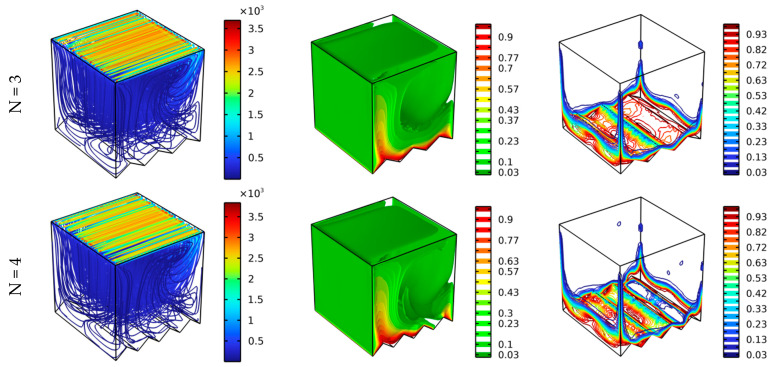
Velocity pathlines, isotherm, and total entropy at different scenarios for Ha = 0, Da = 10^−2^, φ = 0.04, and Re = 100.

Figure 5 shows the impact of the number of zigzags of the lower wall of the room on the distribution pattern of the pathlines, isotherms, and total entropy generation for Re = 100, Ha = 0, and Da = 10^−2^. Initially, it is observed that there is a significant effect of the number of zigzags on the displayed elements. Through Figure 5, it is clearly shown that the movement of the flow inside the container, as well as the temperature gradient near the bottom wall, are very significant for the number of zigzags (N = 2). Through this, it can be predicted that the heat transfer is very important when the number of zigzags is (N = 2).

**Figure 6:**
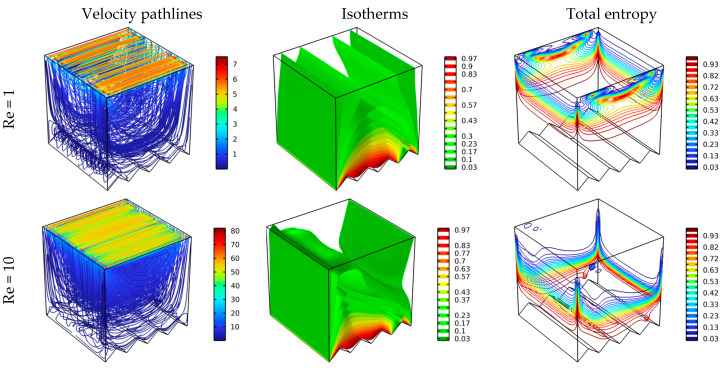

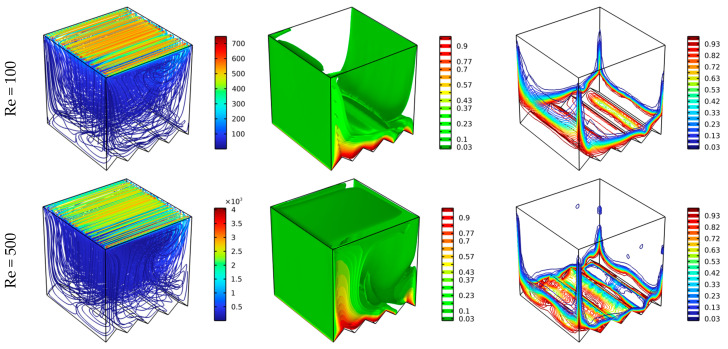
Distribution of velocity pathlines, isotherm, and total entropy for different Re at different scenarios where Ha = 0, Da = 10^−2^, and φ = 0.04.

Figure 6 illustrates the influence of the speed of the movement of the upper wall on the velocity pathlines, isotherms, and total entropy for Da = 10^−2^ and Ha = 0. The speed of the wall movement is expressed in Reynolds numbers. That is, the higher the value of the Reynolds number, the faster the wall. From Figure 6, it is clear that there is a strong influence of the speed of the wall on the displayed items. Generally, the higher the value of the Reynolds number, the greater the permeability of the fluid movement towards the bottom of the chamber. For the contours of pathlines, a circular flow is formed inside the chamber for Re = 1, and then the movement of fluid particles increases in complexity as the value of Re increases. Of course, the transmission of the movement between the moving upper wall and the fluid layers was done through the physical property of the fluid presented by viscosity.

With regard to total entropy contours, it is noted that the higher the value of the Re number, the maximum values of total entropy generation move towards the bottom of the room. The latter increases the temperature gradient around the bottom wall of the chamber, as indicated by the isotherms. Accordingly, we can conclude that the heat transfer is augmented with the increase in the value of Re.

**Figure 7:**
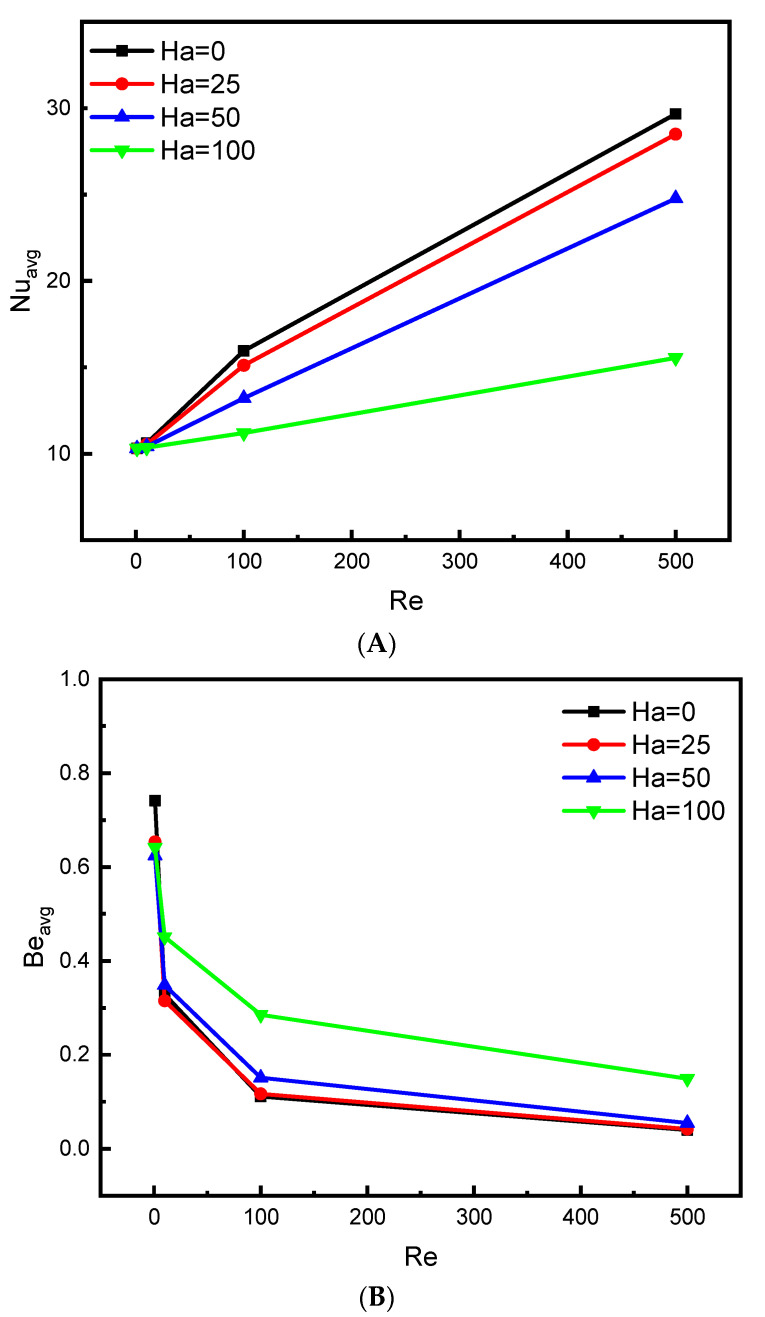
Influence of Ha on (**A**) Nu_avg_ and (**B**) Be_avg_ for Da = 10^−2^ and ϕ=0.04.

Figure 7A,B presents the evolution of the mean values of the Nusselt and Bejan numbers in terms of the Re number with the change in the applied value of the Hartmann number (Ha) for Da = 10^−2^ and N = 4. It is noticed that raising the value of the Re number has a positive impact on the Nu number, while raising the value of the Ha number negatively affects the value of the Nu number. This is, of course, a result of the following: increasing the value of the Reynolds number increases the displacement of the flow inside the container, and the latter improves heat transfer. While raising the Hartmann number increases Lorenz’s effective force that damps the velocity of the flow. Therefore, there is a reduction in heat transfer. Recall that the Bejan number is an expression of the ratio between entropy generation caused by heat transfer and entropy due to friction of flow movement. From Figure 7B, the mean values of the Bejan number (Be) are completely opposite to the values of the Nusselt number. This is due to the fact that the greater the fluid movement, the greater the coefficient of friction, and accordingly, there will be a decrease in Bejan number.

**Figure 8:**
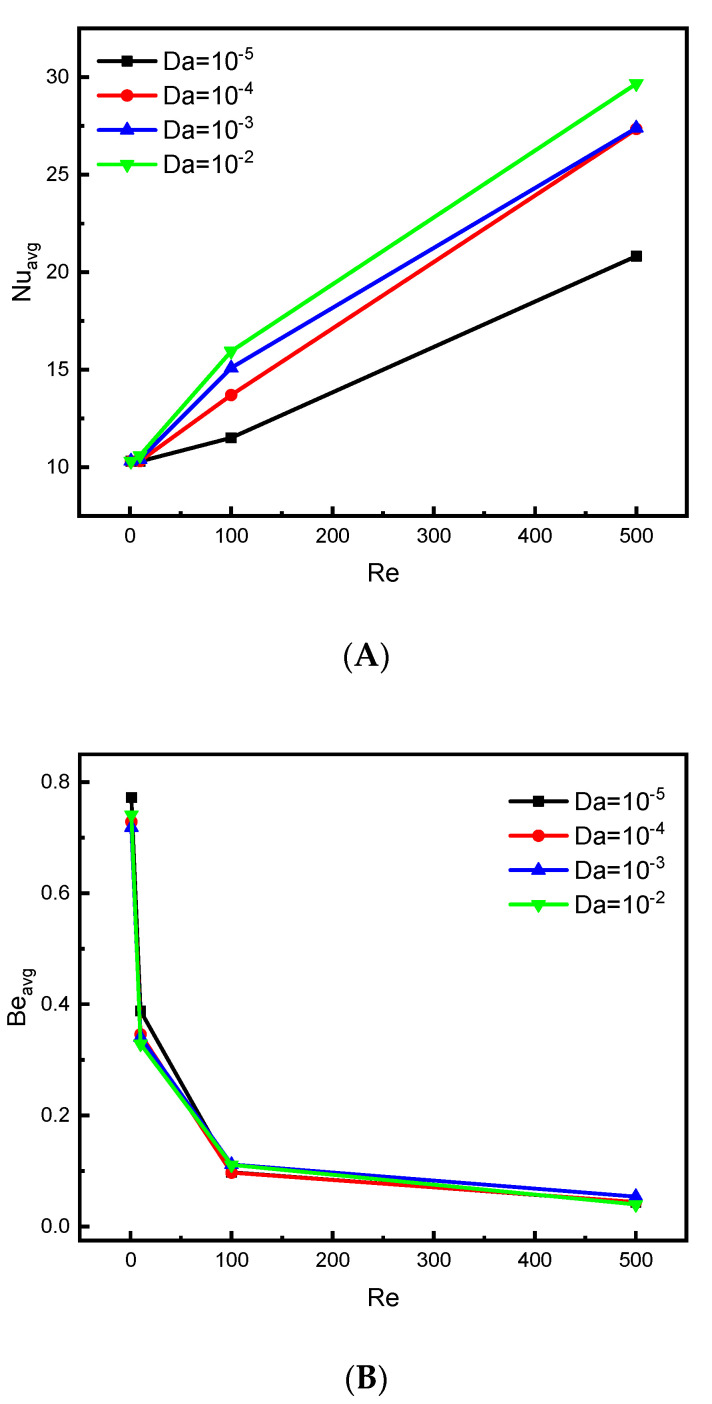
Effect of Da on (**A**) Nu_avg_ and (**B**) Be_avg_ for ϕ=0.04 and Ha = 0.

Figure 8A,B represents the effect of room permeability (Darcy number) on the mean values of the Nusselt and Bejan numbers for various values of Re at Ha = 0 and N = 4. It is clear that the higher value of the Darcy number (Da), the higher value of the Nusselt number (Nu), and this is mainly due to the increase in the permeability of the medium, which facilitates the movement of the flow, which makes the heat transfer process better. There is a slight regression of the values of the Bejan number in terms of the Darcy number. However, the influence of the Reynolds number on the Nu and Be numbers remains the same, as in the previous case.

**Figure 9:**
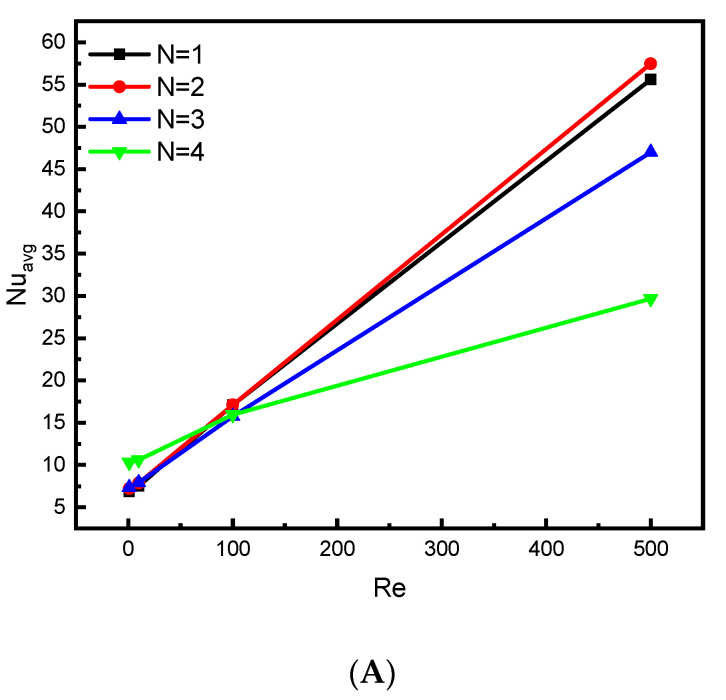

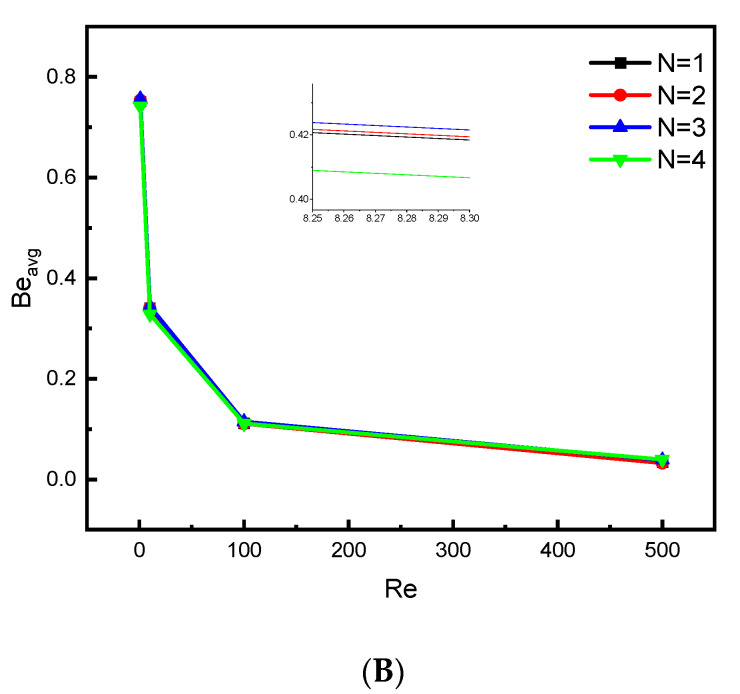
Effect of N on (**A**) Nu_avg_ and (**B**) Be_avg_ for ϕ=0.04 and Ha = 0, and Ha = 0, Da = 10^−2^.

Figure 9A,B presents the influence of the number of lower wall zigzags on the average values of the Nusselt number (Nu) and Bejan number (Be) for Ha = 0 and Da = 10^−2^. The number of zigzags in the bottom wall of the container changes the course of flow, which affects the heat transfer and total entropy generation. Figure 9A proves that the Reynolds number positively affects the Nusselt number for all values of the number of zigzags. In addition, when the value of the Reynolds number is less than 100, meaning that the buoyancy-driven flow is dominant, the values of the Nusselt number are the largest for N = 4. On the other hand, when the Re value is greater than 100, i.e., the forced convection is dominant, the values of the Nusselt number become greater for N = 2, whereas the mean value of the Bejan number is significant when N = 3.

## 5. Conclusions

Through this work, we studied the dynamic and thermal nanofluid patterns inside a three-dimensional chamber. The hybrid nanofluid is of Al_2_O_3_-Cu/water and 4% nanoparticles. The fluid inside the chamber is affected by the medium’s permeability, the horizontal movement of the upper wall, the external magnetic field, the number of zigzags on the bottom side, and the intensity of the thermal buoyancy. The thermal pattern and the dynamic behavior of the hybrid nanofluid under these factors have been studied and understood. The permeability of the medium is well determined in terms of the Darcy number (Da = 10^−5^ to 10^−2^), the velocity of the upper wall was controlled by the Reynolds number (Re = 1 to 500), the intensity of the magnetic system was expressed in terms of a Hartmann number (Ha = 0 to 100), and, finally, the zigzag number was denoted by N (N = 1 to 4). Through the results of the numerical simulations, we were able to conclude the following points:

- Increasing the speed of the horizontal displacement of the upper wall or the permeability of the chamber accelerates the movement of the flow within the room and improves heat transfer.
- Applying the magnetic field and gradually increasing its intensity hinders the movement of the flow particles, and thus negatively affects the thermal transfer.
- The concentration of the total entropy generation depends mainly on the value of the Reynolds number. As the Re value increases, the entropy generation shifts downward.
- The mean value of the number (Nu) is more significant for N = 4 in the case where the natural convection is predominant and for N = 2 in the case where the forced convection is predominant.

## Figures and Tables

**Table 1:** Thermophysical properties of solid nanoparticles (Al_2_O_3_ and Cu base liquid (water) [33,34].

Thermophysical Properties	$Al_{2}O_{3}$	Cu	Water
Density [ $\rho \left(\mathrm{kg}/m^{3}\right)$ **]**	3970	8933	997.1
Specific heat [ $C_{p}\left(J/kgK\right)]$	765	385	4179
Thermal conductivity $\left[k\left(\frac{W}{mK}\right)\right]$	40	400	0.613
Electrical conductivity [ $\sigma \left(S/m\right)]$	$3.69\times 10^{7}$	$5.96\times 10^{7}$	0.05

**Table 2:** Nuavg and Beavg for the different mesh sizes.

No. of Grid Elements	6287	59,960	159,022	511,449	2,163,030
Nu_avg_	15,555	15,542	15,549	15,548	15,548
Be_avg_	0.14927	0.14852	0.14832	0.14832	0.14832

## Data Availability

The results of this study are available only within the paper to support the data.
